# How antibodies alter the cell entry pathway of dengue virus particles in macrophages

**DOI:** 10.1038/srep28768

**Published:** 2016-07-07

**Authors:** Nilda V. Ayala-Nunez, Tabitha E. Hoornweg, Denise P.I. van de Pol, Klaas A. Sjollema, Jacky Flipse, Hilde M. van der Schaar, Jolanda M. Smit

**Affiliations:** 1 Dept. of Medical Microbiology, University of Groningen, University Medical Center Groningen, Groningen, The Netherlands; 2 Dept. of Cell Biology, University of Groningen, University Medical Center Groningen, Groningen, The Netherlands; 3 Dept. of Infectious Diseases & Immunology, Virology Division, Faculty of Veterinary Medicine, Utrecht University, Utrecht, The Netherlands

## Abstract

Antibody-dependent enhancement of dengue virus (DENV) infection plays an important role in the exacerbation of DENV-induced disease. To understand how antibodies influence the fate of DENV particles, we explored the cell entry pathway of DENV in the absence and presence of antibodies in macrophage-like P388D1 cells. Recent studies unraveled that both mature and immature DENV particles contribute to ADE, hence, both particles were studied. We observed that antibody-opsonized DENV enters P388D1 cells through a different pathway than non-opsonized DENV. Antibody-mediated DENV entry was dependent on FcγRs, pH, Eps15, dynamin, actin, PI3K, Rab5, and Rab7. In the absence of antibodies, DENV cell entry was FcγR, PI3K, and Rab5-independent. Live-cell imaging of fluorescently-labeled particles revealed that actin-mediated membrane protrusions facilitate virus uptake. In fact, actin protrusions were found to actively search and capture antibody-bound virus particles distantly located from the cell body, a phenomenon that is not observed in the absence of antibodies. Overall, similar results were seen for antibody-opsonized standard and antibody-bound immature DENV preparations, indicating that the maturation status of the virus does not control the entry pathway. Collectively, our findings suggest that antibodies alter the cell entry pathway of DENV and trigger a novel mechanism of initial virus-cell contact.

Dengue is the most common arthropod-borne viral infection in humans. There are four dengue virus serotypes (DENV1-4) and these cause around 390 million human infections worldwide each year^1^. Approximately 500,000 to 1,000,000 individuals develop severe disease, presenting symptoms like plasma leakage, fluid accumulation, respiratory distress, severe bleeding, and organ impairment^2^. Severe dengue is predominantly seen in infants with declining levels of maternal antibodies and in individuals experiencing a heterologous secondary DENV infection^3^. These observations indicate that pre-existing antibodies are a risk factor for severe disease and led to the well-known hypothesis of antibody-dependent enhancement (ADE) of DENV infection^3^. It is hypothesized that pre-existing cross-reactive DENV antibodies positively influence the infectious properties of the virus^4^. As a consequence, the total infected cell mass increases and this triggers an imbalanced immune response leading to severe disease^4^. It is, however, not completely understood how the antibodies influence DENV infectivity.

DENV infection is mediated by the envelope (E) glycoprotein and involves three important steps: (1) receptor binding, (2) internalization into the host cell, and (3) membrane fusion^5^. DENV E was shown to interact with a wide range of receptor molecules, including C-type lectins, TIM and TAM receptors, and sulfated glycosaminoglycans (GAGs)^6^. Upon virus-receptor binding, DENV particles predominantly enter the cell via clathrin-mediated endocytosis^7^,^8^,^9^. The route of entry is however cell- and virus strain-specific^10^. Membrane fusion typically occurs from within late endosomes, where low pH and anionic lipids trigger conformational changes in the E glycoprotein to mediate membrane fusion^7^,^8^,^9^,^11^. DENV infects a variety of human cells, but cells of the monocyte lineage, like macrophages and dendritic cells, are considered the major target cells for DENV replication^3^.

DENV infectivity is controlled by the viral precursor membrane (prM) protein^12^,^13^,^14^,^15^. Within infected cells, prM has been shown to stabilize the E protein thereby preventing premature conformational changes within E during transit through the acidic Trans-Golgi network (TGN)^12^. Prior to the release of progeny virions, prM is cleaved into M and a pr peptide. This cleavage reaction is however rather inefficient as DENV-infected cells are known to secrete a heterogeneous population of particles, ranging from mature M-containing viruses to fully immature prM-containing viruses^14^. Mature virions are considered to represent the infectious form of the virus. Fully immature particles, on the other hand, are essentially non-infectious in cells lacking DC-SIGN^12^,^13^. Basal low level infectivity of prMDENV was seen in cells expressing DC-SIGN^16^. The threshold of prM cleavage that is required for infectivity is currently unknown, although it is clear that not all prM proteins have to be cleaved for infectivity.

Interestingly, antibodies have been observed to stimulate infectivity of both mature and immature virions, indicating that all particles contribute to ADE of DENV infection^3^,^17^,^18^. All DENV antibodies identified to date can facilitate ADE of DENV infection: enhancement is seen when the antibody concentration falls below the threshold required for virus neutralization^19^. During infection, DENV-antibody complexes are targeted to Fc-γ-receptor (FcγR) bearing cells and upon interaction of the antibodies with FcγR the virion is internalized in the cell. The importance of FcγRs in ADE has been confirmed *in vitro* and *in vivo*^17^,^20^,^21^. Furthermore, the infectivity of antibody-opsonized immature DENV was found to be dependent on cellular furin activity, suggesting that immature virions mature upon virus entry^17^. An early study suggested that antibody-opsonized virions enter the cell via phagocytosis^22^. This was based on the observation that the internalization rate of antibody-opsonized virions resembled the rate of phagocytosis in macrophages^22^,^23^. However, no details are known about the exact cell entry route of antibody-opsonized DENV in FcγR-bearing cells.

In this study, we unraveled the DENV cell entry pathway in the absence and presence of enhancing concentrations of antibodies in macrophage-like P388D1 cells. Virus cell entry was analyzed by (*i*) studying the dynamics of viral entry with single-particle tracking, (*ii*) examining the effect of a variety of endocytic pharmacological inhibitors on viral fusion, (*iii*) imaging – in real time – the interaction of the actin cytoskeleton with fluorescently labeled single virus particles, and (*iv*) exploring the Ab-DENV intracellular pathway by using Rab mutants. Furthermore, to address the role of prM on virus cell entry, we used both antibody-opsonized mature and immature DENV particles.

## Results

### Dynamics of antibody-mediated DENV cell entry

Antibody-mediated cell entry of DENV was investigated by use of human monoclonal antibodies mAb #753 C6 and mAb #3-147, which recognize the E and prM proteins respectively^18^,^24^. Standard DENV (stdDENV) was produced on C6/36 mosquito cells and immature DENV (prMDENV) was cultivated on furin-deficient LoVo cells, as described by us before^13^. LoVo-derived DENV contains high levels of prM (on average 94%) in the viral membrane (13, Fig. S1). The particle-to-PFU ratio of prMDENV preparations used for microscopy was 7 × 10^7^ (Table S1) and at least 100,000-fold reduced compared to that of stdDENV. This is in line with previous studies^13^,^17^ and shows that prMDENV is essentially non-infectious in BHK21 clone 15 cells.

After the initial characterization of the virus preparations, we determined the enhancing profile of the mAbs on murine macrophage-like P388D1 cells. We used P388D1 cells since they express FcγRIII (CD16), FcγRII (CD32), and FcγRI (CD64)^25^,^26^ and are known to support ADE of DENV^27^,^28^. Murine FcγRs are considered to be structurally related to human FcγRs^29^, hence, human antibodies are recognized by P388D1 cells. Prior to infection, mAb #753 C6 was complexed to stdDENV and mAb #3–147 was incubated with prMDENV, as described in the methods section. Maximum enhancement of stdDENV infection was obtained at a mAb concentration of 40 and 400 ng/mL (Fig. S2A). In case of mAb #3–147, optimal ADE of prMDENV was reached at 0.1 ng/mL (Fig. S2B). In agreement with previous studies^13^, we found that prMDENV is non-infectious in macrophage cells in the absence of antibodies (Fig. S2B).

**Figure 1:**
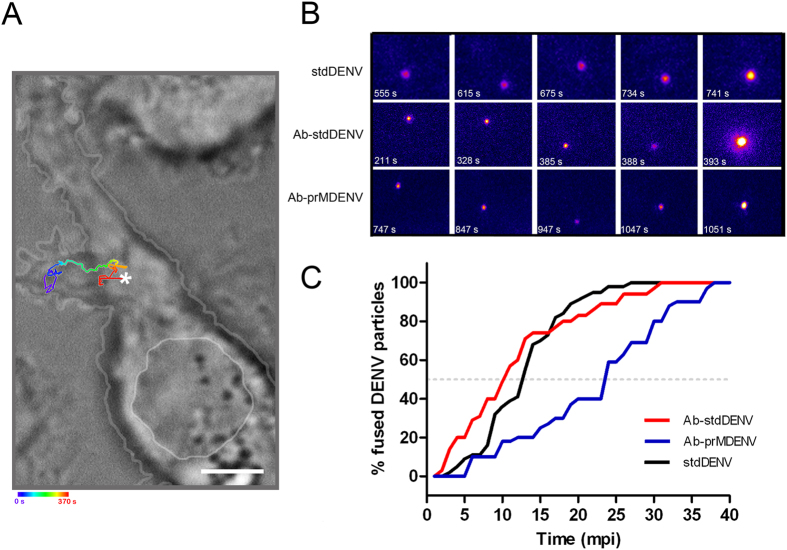
Kinetics of antibody-opsonized DENV entry in macrophages. Single-particle tracking of DiD-labeled Ab-stdDENV, Ab-prMDENV and stdDENV was performed in P388D1 cells at 1 frame per second. (**A**) Cell image obtained with DIC optics showing a trajectory of a single DiD-labeled Ab-stdDENV particle. The white arterisk represents the membrane fusion site. Scale bar: 2 μm. (**B**) Snapshots of a DiD-labeled stdDENV, Ab-stdDENV, and Ab-prMDENV particle at different time points post-infection. Membrane fusion is observed as a sudden increase in fluorescence intensity. The image was artificially modified to show the differences in fluorescence intensity, from low (purple) to high (yellow) intensity. (**C**) The percentage of fused virus particles calculated as a function of time. In total 35 trajectories were analyzed for Ab-stdDENV, 37 for Ab-prMDENV, and 36 for stdDENV. The time point of membrane fusion was defined as the moment when the DiD intensity doubles.

Next, we performed single virus tracking to investigate if antibodies influence the dynamics of viral entry. Single DENV particles were visualized by DiD labeling, as described by us before^7^. The DiD probe was added to purified DENV at a concentration of 2 nmol per 5 × 10^9^ virus particles. At this ratio, a uniformly labeled DENV preparation was seen in the absence and presence of antibodies (Fig. S3). Approximately 75% of the DiD spots had a fluorescence intensity below 40 arbitrary units (a.u.) (Fig. S3C/D). Only particles with a fluorescence intensity lower than 40 a.u. were selected for further analysis as these likely represent single virions. Membrane fusion is detected as a sudden increase in DiD fluorescence intensity due to dilution of the probe into the endosomal membrane^7^. The time point of membrane fusion was defined as the moment when fluorescence at least doubled. DiD-labeled virions were either first pre-incubated with the antibody at concentrations that cause optimal ADE or directly added to P388D1 cells. Subsequently, single-particle tracking of the DiD-labeled virus was performed at a time-lapse of 1 frame per second (37 °C) using epi-fluorescence microscopy. Epi-fluorescence was chosen over confocal microscopy to allow a fast imaging speed. Movies were recorded for 40 min. More than 35 movies were recorded per condition. Figure 1A gives an example of a complete trajectory of an Ab-stdDENV particle in a P388D1 cell. Initially, this particle moves within the cell periphery, and then a rapid movement likely via microtubules (red segment of trajectory in Fig. 1A) is observed prior to membrane fusion. The representative movie of this trajectory is uploaded as Movie S1. Only a limited number of complete trajectories were recorded as in most movies parts of the trajectory were out of focus due to the thickness of P388D1 cells. Therefore, we were not able to quantify the trafficking behavior of DENV cell entry in macrophage-like cells.

In Fig. 1B, snapshots of DiD-labeled particles are shown over time. In these images, fusion is seen at 742, 393 and 1051 seconds post-addition to the cells for stdDENV, Ab-stdDENV and Ab-prMDENV, respectively. Quantitative analysis revealed a considerable delay in the fusion time point of Ab-prMDENV with respect to Ab-stdDENV and stdDENV (Fig. 1C). As illustrated in Fig. 1C, 50% of the Ab-prMDENV particles have fused within 24 min (gray dashed line). On the other hand, however, 50% fusion of Ab-stdDENV and stdDENV particles was seen at 10 min and 13 min respectively, indicating that the presence of prM in the membrane drastically influences the time to membrane fusion.

### Analyzing DENV cell entry by pharmacological inhibitors

**Figure 2:**
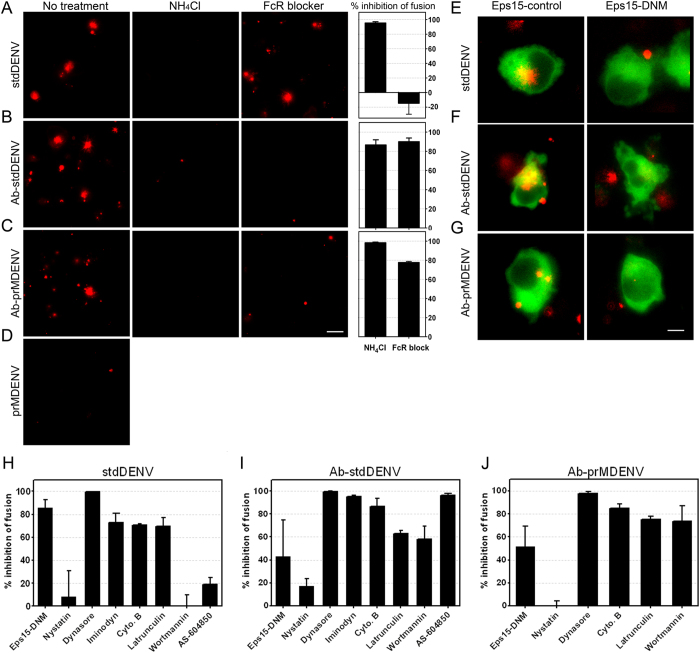
Antibody-opsonized DENV entry inhibition by endocytic inhibitors. DiD-labeled DENV (opsonized and non-opsonized) was added *in situ* to P388D1 cells in the presence or absence of the indicated inhibitors. After 30 min of infection at 37 °C, the cells were washed and snapshots were taken with an oil-immersion 100× objective. (**A–D**) Representative images upon DiD-DENV infection with and without prior treatment of the cells with NH_4_Cl (50 mM) and FcR blocker are shown. Scale bar: 12.5 μm. Fusion inhibition was calculated by analyzing the total extent of membrane fusion of DiD-labeled virus with ImageJ. (**E–G**) Representative images of a fusion assay performed in P388D1 cells electroporated with Eps15-GFP (empty vector D3Δ2 (control) and DNM (E95/295)). Scale bar: 7 μm. (**H–J**) Fusion inhibition of Eps15 and multiple biochemical inhibitors. An end concentration of 50 μM nystatin, 150 μM dynasore, 200 μM iminodyn-22, 15 μM cytochalasin B, 1 μM latrunculin, 2 μM wortmannin, or 30 μM AS-604850 was used. The percentage of fusion inhibition was calculated with respect to the empty vector or non-treated control, respectively. The average of at least three independent experiments is shown. Error bars represent SEM.

We next evaluated the cell entry pathway of DENV in the absence and presence of antibodies by using various inhibitors of known endocytic pathways. The concentration of the inhibitors used was based on successful inhibition of entry of known fluorescently labeled cargo controls, like transferrin, dextran and cholera toxin B (Fig. S4A-E). Furthermore, the cellular metabolic activity – an indicator of cell viability – had to be higher than 75% at the effective concentration, as defined by MTT assays (Fig. S4F). The effect of the inhibitor on DENV cell entry was measured by use of a fluorescence microscopy-based fusion assay^30^. The novelty of this approach lies within the fact that the inhibitory effect is directly measured at the level of virus entry and membrane fusion, thereby minimizing potential pleotropic effects of the drugs. The extent of membrane fusion was determined at 30 min post-addition of the virus to the cells as the vast majority of the viruses have fused within this time frame (Fig. 1C). At 30 min post-infection, cells were washed and the extent of membrane fusion was determined by measuring the total fluorescent signal in 15 randomly selected imaging fields, as described in the methods section. Figure 2A–D shows representative images of the fusion assays in P338D1 cells. As expected^17^, no fusion was seen for prMDENV in the absence of antibodies. Furthermore and in line with literature^5^, no fusion was seen in cells treated with ammonium chloride (NH_4_Cl), a compound known to neutralize the pH of intracellular compartments. We also noted an overall increase in fluorescence intensity when comparing the images from stdDENV and Ab-stdDENV. Indeed, subsequent quantification of the fluorescence intensity in three independent experiments revealed that membrane fusion activity is approximately 3.5-fold increased under conditions of ADE (Fig. S5A). The observed increase in membrane fusion activity is mainly caused by an increase in the number of fusion-positive cells (2.3 fold, Fig S5B). To address if enhanced fusion is due to increased binding of Ab-DENV complexes to cells we next determined the number of bound particles by qRT-PCR (Fig. S5C). The results show that the enhanced membrane fusion activity as observed under ADE conditions can be largely explained by increased binding and entry of DENV-immune complexes to P388D1 cells. At ADE conditions, binding is facilitated through antibody-Fc receptor interactions as both viral infectivity (Fig. S6A) and membrane fusion activity (Fig. 2B,C) is severely impaired upon treatment of the cells with anti-FcγR CD16/CD32 blocker.

Initially, we investigated the role of clathrin and caveolae in DENV cell entry in the absence and presence of antibodies. At first, we tried to use chlorpromazine and pitstop2, two well-known inhibitors of clathrin-mediated endocytosis. However, these compounds were quite toxic, and at non-toxic concentrations these compounds were unable to sufficiently block transferrin uptake (Fig. S4). Thereafter, we tested a dominant negative mutant (DNM) of Eps15 (E95/295), a protein that is described to be present in clathrin-coated pits and is important in clathrin-mediated endocytosis^31^. As seen in Fig. 2E–J, the Eps15-DNM inhibited fusion in all three infection conditions, albeit to a different extent. The most extensive inhibition (>80%) was seen following infection with stdDENV in the absence of antibodies (Fig. 2E,H). Mediocre inhibition was seen in case of Ab-stdDENV (~40%) and Ab-prMDENV (~45%). Treatment of cells with nystatin, a cholesterol binding agent, leads to a complete ablation of morphologically identifiable caveolae^32^. We observed that nystatin treatment had no influence on stdDENV, Ab-stdDENV or Ab-prMDENV fusion (Fig. 2H–J), which suggests that entry of DENV with and without Abs is caveolae-independent.

Thereafter, the role of dynamin and actin was measured. Dynamin, a small GTPase, has a critical role in membrane fission events during clathrin- and caveolae-mediated endocytosis and phagocytosis^33^,^34^. Actin is involved in numerous endocytic pathways, like clathrin-mediated endocytosis, macropinocytosis, caveolae-mediated endocytosis, and phagocytosis^35^. Though the actin cytoskeleton function is variable between distinct endocytic pathways^35^. Treatment of P388D1 cells with dynasore, a compound that interferes with the GTPase activity of dynamin1 and dynamin2^36^, abolished membrane fusion activity and infectivity of DENV irrespective of the maturation status and with and without prior opsonization with antibodies (Fig. 2H–J and S6B). Furthermore, iminodyn-22, another dynamin inhibitor, reduced the membrane fusion extent of stdDENV with 75% and for Ab-stdDENV with 95%. Disruption of actin filaments with cytochalasin B and latrunculin was found to reduce fusion of Ab-stdDENV, Ab-prMDENV and stdDENV (Fig. 2H–J). Inhibition of PI3K, a key coordinator in actin remodeling, through wortmannin specifically reduced membrane fusion activity of Ab-stdDENV and Ab-prMDENV (Fig. 2H–J). The importance of PI3K in Ab-stdDENV cell entry was confirmed by the use of AS-604850 (Fig. 2I). Wortmannin and AS-604850 had little to no effect on stdDENV fusion (Fig. 2H). These results demonstrate that actin and the regulators of actin remodeling play a critical role during Ab-DENV endocytosis. The different response to PI3K inhibitors is consistent with a distinct DENV cell entry mechanism in the presence and absence of antibodies.

### Ab-DENV activates extensive ruffling of the cell membrane via FcγRs

**Figure 3:**
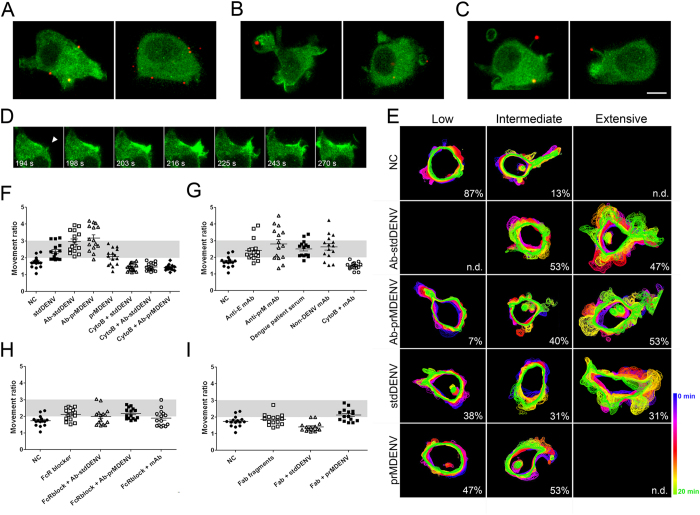
Ab-DENV induction of actin cytoskeleton rearrangement via FcγRs. DiD-labeled virus (Ab-stdDENV, Ab-prMDENV and stdDENV) was added to eYFP-Actin-expressing P388D1 cells. The cells were kept at 37 °C for live-cell imaging with a spinning disk confocal microscope. Ab-DENV was observed to localize to the (**A**) cell body, (**B**) membrane ruffles and (**C**) actin filopodia during entry. Scale bar: 7 μm. (**D**) Time series of antibody-opsonized DENV induced cell membrane ruffling. The white arrowhead indicates the place where the ruffle starts. (**E–I**) Cell movement was quantified over time upon the indicated treatments. (**E**) Representative images of P388D1 cells under the indicated conditions are shown as the overlays of the cell body outlines of a time-lapse of 20 min. (**F–I**) The Movement ratio (MR) quantifies the extent of movement of one cell over a frame sequence. See Fig. S7 for more details on how to calculate it. Based on the MR, the movement response was classified as Low (MR < 2), Intermediate (MR = 2–3) or Extensive (MR > 3). For each condition, 15 cells were used for analysis. The gray area indicates the intermediate response. The proportion of cells showing Low, Intermediate, and Extensive response is indicated at the right corner of (**E**).

The actin cytoskeleton has been observed to support different processes related to endocytosis, like the invagination of membrane segments into the cytoplasm, elongation of invaginations, and protrusions in the form of filopodia and ruffles^35^,^37^. In order to study how DENV particles interact with the actin cytoskeleton, we performed live-cell imaging in eYFP-Actin expressing P388D1 cells. Interestingly, dramatic changes in cell morphology and actin redistribution were observed upon infection with DiD-labeled Ab-stdDENV and Ab-prMDENV (Fig. 3). Ab-stdDENV and Ab-prMDENV particles were not only seen at the cell body (Fig. 3A), but also associated with membrane ruffles (Fig. 3B) and filopodia-like structures (Fig. 3C). Ruffles were observed across the cell membrane as sheet-like protrusive structures with a heterogeneous morphology (Fig. 3D). The extensive actin-driven membrane ruffling of the cell is illustrated by Movies S2 and S3. In the absence of any added stimuli (negative control), the eYFP-Actin expressing macrophages are constitutively active (as reported by Flannagan *et al*.^38^), but do not show widespread ruffling of the membrane (Movie S4). Ruffling induced by Ab-stdDENV and Ab-prMDENV is initiated at 3 to 5 minutes post-infection (mpi) (Movies S5 and S6). Movie S5 shows eYFP-Actin expressing cells during the first 4.5 minutes following infection with Ab-stdDENV. DiD-labeled particles are visible, but not yet bound to the cell membrane. A constitutive movement of the cell body can be seen. Movie S6 shows the same group of cells at 5 mpi. A widespread and highly active ruffling of the cells is evident.

But what is triggering membrane ruffling? Is it the immune complex, the virus or the antibody? To address this issue, we first developed an ImageJ-based method to quantify the extent of cell ruffling in one cell. With this method, we calculated the “Movement ratio” (MR) by dividing the “total area” covered by a moving cell for the whole sequence, with respect to the smallest surface area occupied by the same cell in the same time series (explained in detail in Fig. S7). Based on the resulting Movement ratio, cells were classified in three groups: Low (MR < 2), Intermediate (MR = 2–3), and Extensive (MR > 3). To visually illustrate the results, the projections of the cell body outlines of a time-lapse of 20 min are shown in Fig. 3E. No differences were observed between the ruffling response activated by Ab-stdDENV (Movie S2) and Ab-prMDENV (Movie S3). Extensive membrane ruffling is observed in approximately 50% of the cells infected with Ab-stdDENV (47%) and Ab-prMDENV (53%) (Fig. 3E,F). Ruffling is also observed in cells infected with stdDENV and prMDENV in the absence of antibodies, but this ruffling is less extensive compared to Ab-stdDENV and Ab-prMDENV (Fig. 3E,F). Cytochalasin B prevented membrane ruffling in all conditions (MR < 2). The addition of soluble anti-E and anti-prM antibodies to the cells also induced membrane ruffling, albeit to a somewhat lower extent than antibody-opsonized DENV (Fig. 3G). Similar results were found upon addition of dengue patient immune serum and an non-DENV murine IgG antibody. These results prompted us to further study the role of antibodies in ruffling induction of the cell membrane. The use of a FcγR blocker (anti-CD16/CD32) prevented ruffling induced by Ab-stdDENV, Ab-prMDENV and by soluble anti-E mAbs (Fig. 3H). In addition, we observed that Fab fragments with and without prior opsonization to stdDENV and prMDENV particles do not induce extensive ruffling (Fig. 3I). Taken together, these results indicate that activation of membrane ruffling in macrophages by Ab-stdDENV and Ab-prMDENV is likely triggered through the interaction of the antibodies with FcγRs expressed at the cell surface.

### Antibody-mediated DENV entry is not mediated by macropinocytosis

**Figure 4:**
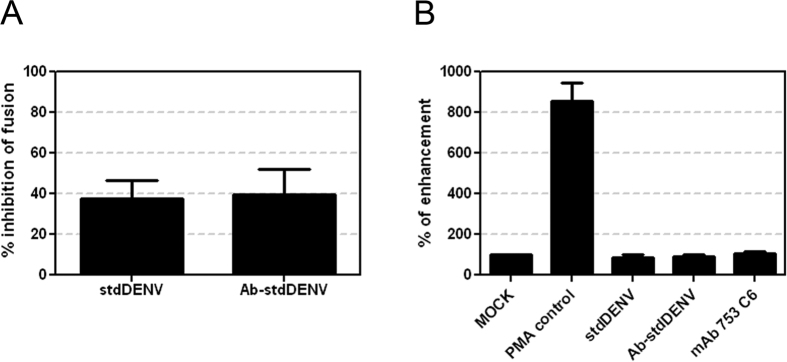
Role of antibody-induced macropinocytosis in virus internalization. (**A**) P388D1 cells were treated with EIPA (25 μM) for 1 h at 37 °C. Cells were infected with DiD-labeled stdDENV and Ab-stdDENV and analyzed as in Fig. 2. (**B**) Fluid phase uptake of Dextran-TxRd upon addition of stdDENV, Ab-stdDENV or mAb # 753 C6. PMA (60 ng/ml) was added as a positive control. All treatments were added 15 min before addition of Dextran-TxRd. Uptake of Dextran (0.4 mg/ml) was allowed for 30 min at 37 °C. The average of five independent experiments is shown. Error bars represent SEM.

Ruffling, the involvement of actin, and the role of PI3K is suggestive for Ab-stdDENV cell entry via macropinocytosis. Macropinocytosis has been suggested as an alternative DENV cell entry pathway in HepG2 cells and was found to be important for several other viruses^9^,^39^. Macropinocytosis is an actin-dependent process characterized by the formation of macropinosomes, which are structures derived from the eukaryotic plasma membranes ranging in size from 0.2 to 10 μm that are used to engulf extracellular fluid for cellular uptake^40^. The effect of amiloride derivative EIPA on virus cell entry is often used to study the importance of macropinocytosis. EIPA has been described to inhibit Na^+^/H^+^ exchanger (NHE) activity thereby modulating Rho GTPases and interfering with macropinosome formation^41^. EIPA inhibited membrane fusion activity of Ab-stdDENV and stdDENV albeit with a rather low efficiency (Fig. 4A). The weak inhibitory effect of EIPA combined with the notion that NHEs can also be found in membranes of other intracellular vesicles^42^, thereby potentially interfering with other pH-dependent pathways, makes it difficult to interpret this data. Therefore, we next investigated another hallmark of macropinocytosis: increased fluid uptake. Figure 4B shows that there is no increased fluid uptake during infection with stdDENV, Ab-stdDENV and upon the addition of soluble antibody, whereas a marked increase in fluid uptake was seen upon addition of the known macropinocytosis inducer PMA. Furthermore, upon close examination of the recorded movies, only very few ruffles were seen that close to form vesicles and only a minor fraction of these vesicles contained visible Ab-stdDENV (4 out of 50) or Ab-prMDENV (2 out of 50). Taken together, although we noticed extensive actin-mediated ruffling at the plasma membrane and sometimes closure of vesicles, macropinocytosis does not appear to have an important role in DENV cell entry in macrophages.

### Interaction of Ab-opsonized DENV with actin cell-surface protrusions

**Figure 5:**
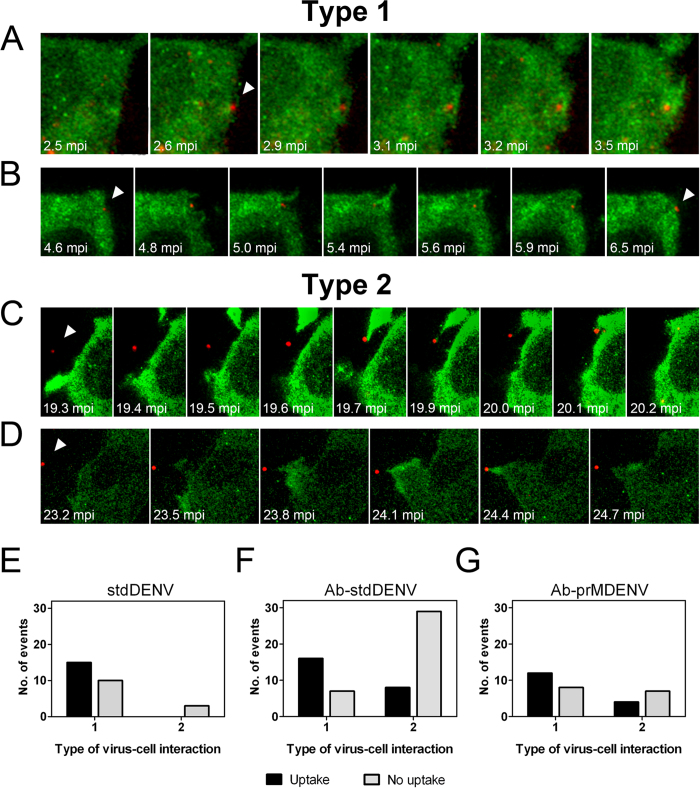
Ab-DENV uptake is mediated by actin protrusions. The experimental set-up is similar as described in the legend to Fig. 3. (**A–D**) Representative montages of alternating time-lapse exposures of DiD-DENV and eYFP-Actin during entry. (**A,B**) Type 1 virus uptake. (**C,D**) Type 2 virus uptake. DiD-virus is shown in red. eYFP-Actin expressing cells are shown in green. White arrowheads point out single viral particles associated with actin structures. (**E–G**) Frequency of Type 1 and type 2 uptake in a total of 60 events.

To better understand how virus particles interact with the plasma membrane and to decipher the role of actin herein, we further analyzed the recorded movies. We observed that many virus particles were indeed internalized through localized cell-surface actin protrusions (Fig. 5). These were not considered as part of the ruffling response described in the previous section, since ruffling was observed as a general response of the complete cell body and these protrusions represent localized reactions to a viral particle.

We observed two types of localized actin-mediated uptake of Ab-DENV. Type 1 implies uptake through actin protrusions that are formed upon direct virus-cell contact (Fig. 5A). In case of Type 2, actin protrusions were generated towards viral particles located away from the cell body. Once the actin-mediated structure reached the virus particle, the protrusion retracts and pulls back the virion to the cell membrane (Fig. 5C and Movie S7).

To evaluate the efficiency of these processes we quantified the number of events by visual examination. Type 1 and Type 2 virus-cell interactions were counted in 73 cells per virus condition and classified as successful or unsuccessful, based on the ability of the actin protrusion to mediate viral uptake. In total, 29 virus-cell interactions were recorded for stdDENV, 60 virus-cell interactions for Ab-stdDENV and 31 for Ab-prMDENV. Representative examples are shown in Fig. 5A–D. Figure 5A shows a successful Type I uptake. The antibody-opsonized DiD-labeled particle is bound to the plasma membrane. At 2.6 mpi, an actin protrusion starts to surround the virus particle and subsequently captures it. Figure 5B shows an unsuccessful Type 1 uptake. This particle is surrounded by actin protrusions but did not end up inside a cellular compartment. A successful Type 2 uptake is illustrated in Fig. 5C. Initially, an antibody-opsonized virus is seen approximately 5 μm away from the cell body. Then, an actin-rich structure protrudes and elongates in the direction of the virus particle. This protrusion reaches and binds to the viral particle, and pulls it back to the cell body. The complete process lasts 54 seconds. This is also visible in Movie S7. Figure 5D presents an unsuccessful Type 2 uptake. Here, actin protrusions are directed towards the virion but fail to pull it back. Since we sometimes observed repeated attempts of the cell to capture the same particle (Movie S8), we believe that Type 2 movement represents a specific direct process to capture virus particles from the extracellular environment. In all conditions, Type 1 appears to be more efficient than Type 2 (Fig. 5E–G). The frequency of successful Type 1 uptake is similar for Ab-stdDENV, Ab-prMDENV and stdDENV (Fig. 5E–G). Interestingly, Type 2 interactions were hardly detected following stdDENV infection in the absence of antibodies. Moreover, the Type 2 interactions that were seen upon stdDENV infection were never successful. Successful Type 2 uptake was however seen in 8 out of 36 (22%) events for Ab-stDENV and 4 out of 11 (36%) for Ab-prMDENV (Fig. 5E,F).

As observed in Fig. 5C, Type 2 uptake is directed specifically against a single virus particle that is not moving and not associated with the plasma membrane or any other visible actin structure. But how is the cell able to sense the presence and position of the virus without having contact with it? Or is the membrane surface area of the cells wider than seen by eYFP-Actin? To address this issue, we stained the membranes of eYFP-Actin expressing cells with the lipophilic probe DiD and used high resolution differential interference contrast (DIC) microscopy to evaluate the membrane surface area of the cells. The DiD signal completely overlaps with eYFP-Actin, indicating that all cellular structures are detected with eYFP-Actin (Fig. S8). At this moment we do not know how cells sense the virions. Importantly, though, successful Type 2 interactions were only observed in case of Ab-stdDENV and Ab-prMDENV and therefore this sensing mechanism is linked to the presence of antibodies (Fig. 5G).

### Endocytic trafficking of antibody-opsonized DENV

**Figure 6:**
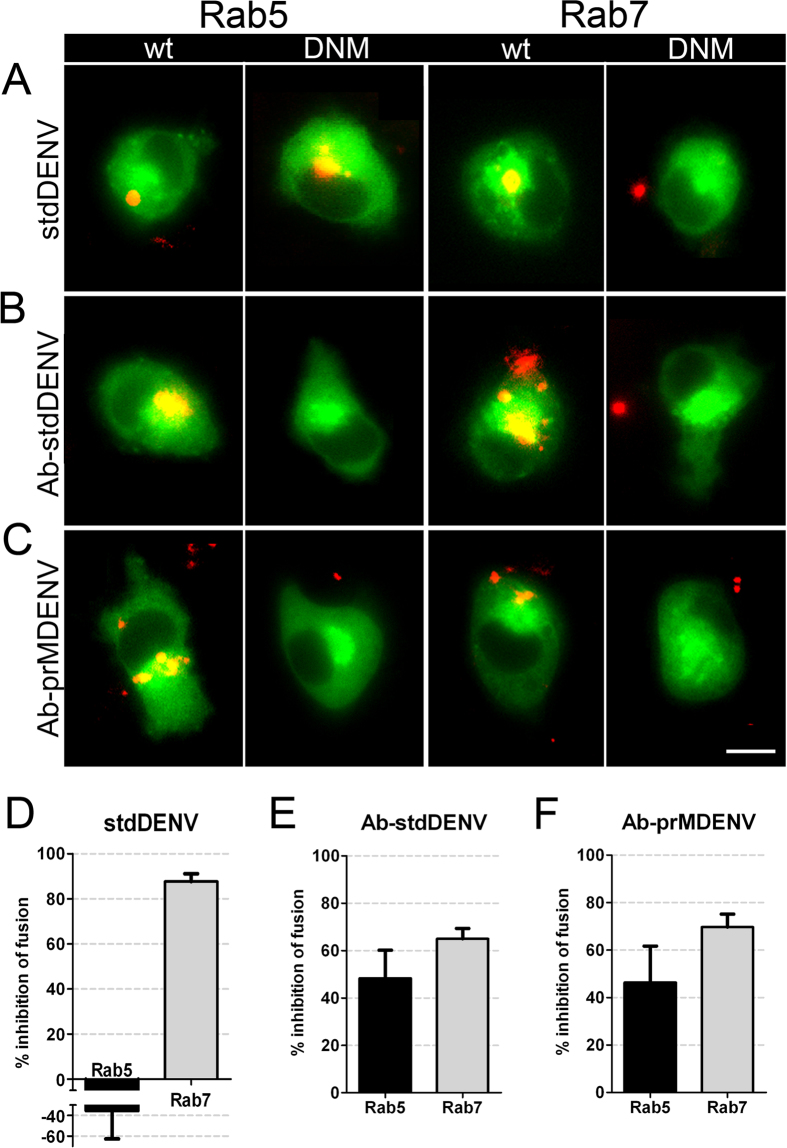
Effect of Rab5 and Rab7 dominant-negative mutants in fusion of Ab-DENV. DiD-labeled DENV (with and without antibodies) was added *in situ* to P388D1 cells electroporated with the indicated plasmids. (**A–C**) Representative examples of membrane fusion. Membrane fusion of DiD-labeled virus is observed as highly fluorescent red puncta in the WT and DNM electroporated cells. All plasmids have GFP as a reporter gene. Scale bar: 7 μm. (**D–F**) Quantification of the effect of the DNM proteins on viral fusion. For analysis, 85 cells positive for the expression of the plasmids were used. The percentage of inhibition was calculated with respect to the WT plasmid control.

After uptake and internalization, most viruses traffic through intracellular vesicles until a compartment is reached where membrane fusion occurs (refer to^43^ for an extensive review). The intracellular compartments hijacked by viruses can be endosomes, phagosomes or pinosomes, all of which have been described to undergo acidification and a maturation process that involves the gain and loss of early/late markers before fusion with lysosomes^39^,^43^. Since trafficking of the different intracellular compartments is controlled by Rab GTPases^44^, we used a set of fluorescently-labeled Rab dominant-negative mutants (DNM) to analyze which of these GTPases is required for viral transport. Fluorescently-labeled dextran was used as a control to evaluate the activity of the DNMs. Figure S9 shows that the overexpression of Rab5 and Rab7 DNMs inhibited uptake of the fluorescently-labeled dextran. DENV membrane fusion activity was reduced in cells transfected with DNM of Rab7 for all experimental conditions (Fig. 6A–C), particularly in the case of stdDENV in the absence of antibodies (88% of inhibition with respect to the WT Rab7). Interestingly, Rab5 was important for Ab-DENV fusion (black bars, Fig. 6D–F), but not for stdDENV. Unfortunately, though, these results could not be confirmed by flow cytometry given the low transfection and infection efficiency in macrophages.

## Discussion

The study presented here aimed to understand how DENV enters macrophages in the absence and presence of antibodies. DENV entry in P388D1 cells was found to be Eps15, dynamin, actin, pH and Rab7-dependent, but FcγR, caveolae, PI3K, macropinosome and Rab5-independent. Antibody-mediated DENV entry, on the other hand was found to be FcγR, dynamin, actin, PI3K, pH, Rab5 and Rab7-dependent, partially dependent on Eps15, and caveolae- and macropinosome-independent. Thus, the route of DENV cell entry in the absence and presence of antibodies can be distinguished on the basis of FcγR, PI3K and Rab5. In addition, a novel mechanism of virus capture was detected during antibody-mediated uptake of DENV particles in P388D1 cells. Furthermore, except for the time to membrane fusion, there were no evident differences between the entry profile of Ab-prMDENV and Ab-stdDENV particles.

Dengue virus cell entry in the absence of antibodies does not fit with a classical textbook view of one endocytic process. The importance of Eps15 and dynamin is suggestive for entry via clathrin-mediated endocytosis although also clathrin-independent pathways have been described to use dynamin and Eps15^31^,^45^. The actin cytoskeleton is important in numerous endocytic and phagocytic processes, but the function of actin within these processes is different^37^. In clathrin-mediated endocytosis, actin has been described to support the process of invagination of the membrane and can help with the elongation and scission of the new vesicle^37^. We observed that actin forms active membrane protrusions that engulf the particles (Type 1), a phenomenon that has been linked to phagocytic processes^46^,^47^. Entry through phagocytosis seems unlikely, however, since PI3K is not required in DENV cell entry and this molecule is generally believed to be important for phagosomal maturation^46^,^48^,^49^. Therefore, we propose that DENV entry in macrophages is via a novel mechanism involving elements of clathrin-mediated endocytosis and phagocytosis. The ability of a virus to use non-classical entry pathways displaying features of multiple established endocytosis pathways has been described before^50^,^51^,^52^. Furthermore, enhanced membrane fusion was seen in Rab5-DNM expressing cells whereas a marked inhibition was noticed in Rab7-DNM cells. This suggests that DENV particles bypass Rab5 organelles and are immediately delivered to Rab7 compartments where membrane fusion occurs. This is in contrast to our earlier results in BS-C-1 cells^7^ and indicates that the endocytic behavior is cell type-specific. Although more experiments are required to confirm this finding, similar results have been described for lymphocytic choriomeningitis virus^51^. The authors suggested that this atypical transport behavior may serve to internalize plasma membrane molecules and bound ligands that need to avoid recycling regulated by Rab5 early endosomes^51^. The conclusion that DENV fuses from within Rab7 compartments is in agreement with our earlier data^7^ and is in line with the described lipid dependence for DENV fusion^11^.

The internalization profile of antibody-opsonized DENV is similar to stdDENV entry except for the role of FcγRs and PI3K. This implies that antibodies influence the fate of DENV cell entry. FcγRs^46^, dynamin^53^, active formation of actin protrusions^46^, and PI3K^54^ have been prior linked to phagocytosis. Furthermore, Type I uptake looks like a phagocytic cup. Therefore, we propose that cell entry of antibody-opsonized DENV occurs via phagocytosis, which is in line with the conclusions of Halstead *et al*.^22^. Inhibition of Eps15 partially inhibited antibody-opsonized DENV entry in macrophages. This can be explained by phagocytosis, as different elements of the clathrin machinery, like AP-2, have been located in the outer layer of phagosomes and play a role in phagosomal maturation^55^. Phagocytosis-like entry may seem unexpected given the relatively small size of Ab-DENV complexes. Indeed, it is generally believed that phagocytosis is meant for uptake of particles larger than 0.5 μm^46^. Yet, phagocytosis-like pathways have been described for viruses smaller than 0.5 μm, like Adenovirus type 2 (90–100 nm)^56^ and HSV-1 (200 nm)^57^. Hence, the size of the particles is not the only determinant needed to trigger phagocytosis. Upon phagocytosis, the antibody-opsonized particles traffic through Rab5 and Rab7 organelles and both early and late phagosomes are required for entry and membrane fusion in P388D1 macrophages. Given the pH and lipid requirements for fusion, we anticipate that the virus predominantly fuses from within late phagosomal compartments.

A surprising novel finding was Type 2 uptake of antibody-opsonized stdDENV and prMDENV. To our knowledge, this is the first study that describes the possibility of a cell to actively search and capture a virus particle from the environment. But how is the cell able to detect virus particles located away from its main body? A possible explanation is chemotaxis. Antibodies are known to associate and dissociate from their antigen over time. It is tempting to speculate that the dissociated antibodies leak away from the complex and serve as a chemotactic stimulus that macrophages can sense. Indeed, successful Type 2 uptake was not observed with stdDENV in the absence of antibodies. Thus, antibodies not only flag the virus as a foreign antigen that needs to be degraded, they may also induce an active capturing mechanism in macrophage cells. In this sense, Type 2 is a remarkable example of how viruses could utilize pathogen-sensing functions of macrophages.

Extensive membrane ruffling was observed upon DENV infection in presence of antibodies. Most of these ruffles did not close to form macropinosomes and no enhanced fluid uptake was seen. This shows that extensive membrane ruffling is not necessarily linked to the process of macropinocytosis. Indeed, membrane ruffling has also been linked to receptor internalization, preparation of cell motility, and cell migration^58^. Future studies should reveal whether the observed membrane ruffling, which is triggered by antibody-Fc receptor interaction, indeed reflects the initiation of cell motility and/or migration.

We observed that the fusion time point of DiD-labeled Ab-prMDENV particles is approximately two times delayed compared to Ab-stdDENV. Yet, there were no clear differences observed in the entry profile, indicating that the maturation status of the virus influences the time to membrane fusion but not the entry pathway itself. Previous studies showed that immature DENV has to undergo a furin-dependent maturation process prior to membrane fusion^17^. It is known that progeny virions release the pr peptide upon secretion of the particle to the extracellular pH neutral milieu. During infection, it is hypothesized that the lower pH of late phagosomal compartments – when compared to the luminal pH of vesicles within the Golgi apparatus – is sufficient to release the pr peptide from the virion^59^. Hence, we postulate that the additional time required to initiate membrane fusion reflects the processes involved in virion maturation rather than hijacking a distinct route of cell entry.

DENV-immune complexes bound more efficiently to P388D1 cells than DENV particles in the absence of antibodies. In primary macrophages, however, no increase in virus cell binding and entry was observed under ADE conditions (unpublished results/accompanying manuscript). These contradicting results may reflect differences in virus receptor and Fc receptor expression levels^60^,^61^. Indeed, in the absence of antibodies, the number of bound particles was much lower in P388D1 cells than in primary macrophages. On the other hand, however, comparable numbers of bound particles were seen in P388D1 cells and primary macrophages under ADE conditions. This suggests that P388D1 cells express lower levels of virus receptors than primary macrophages. The relative low binding efficiency of DENV to P388D1 cells might also explain why a more robust ADE effect is seen in these cells. Indeed, previous studies on immature dendritic cells revealed that the expression level of the virus receptor DC-SIGN is inversely correlated with ADE^60^.

It is important to mention that the antibody/FcR-mediated entry pathway by itself does not explain ADE. Under neutralizing conditions, virions will also enter macrophages via an antibody-mediated pathway^62^. Indeed, FcγRs have been shown to be important in antibody-mediated neutralization as well as antibody-mediated enhancement of infection^3^,^62^,^63^. Hence, it is likely that a similar entry pathway is used under neutralizing and enhancing conditions. What then determines the enhancing or neutralizing capacity of the antibody? We and others have suggested that ADE is controlled at the level of membrane fusion^61^,^63^. FcγRIIBFusion and thus ADE may occur when antibodies dissociate from the virion during entry or bind with low occupancy thereby allowing unoccupied E proteins to initiate fusion. At very high concentrations of neutralizing antibodies, DENV-Ab aggregates are formed and inhibit phagocytosis through crosslinking of FcγRIIB^62^, which suggests that depending on the antibody concentration of neutralizing antibodies distinct mechanisms can occur.

In conclusion, our results provide evidence for a novel antibody-mediated mechanism meant to regulate the uptake and internalization of DENV particles in macrophages. The overall entry pattern of Ab-stdDENV and Ab-prMDENV was similar, indicating that the antibody dictates the route of entry and not the virion. This data not only elucidates how antibodies alter the cell entry pathway of DENV particles in macrophages, but also shows that Ab-virus complexes can take advantage of an active pathogen-sensing mechanism of macrophages. It would be interesting to establish whether the intervention of these entry pathways may be useful in the design of antiviral drugs that can be used in dengue severe disease.

## Methods

### Cells and viruses

Murine macrophage P388D1 cells were kindly provided by Dr. Michael Diamond (Washington University in St. Louis, USA), they are also available through ATCC (No. CCL-46). P388D1 cells were maintained in DMEM (PAA) supplemented with 10% FBS, penicillin (100 U/mL), streptomycin (100 μg/mL), sodium bicarbonate (Invitrogen, 7.5% solution) and 1.0 mM sodium pyruvate (Gibco). Baby hamster kidney clone 15 (BHK-15) cells (gift from Dr. Richard Kuhn, Purdue University, USA) were cultured in 1x high glucose, L-glutamine-enriched DMEM with 10% FBS, penicillin (100 U/mL), and streptomycin (100 μg/mL). *Aedes albopictus* C6/36 cells (also obtained from Dr. Richard Kuhn, ATCC No. CCL-1660) were maintained in MEM (Gibco) supplemented with 10% FBS, 25 mM HEPES, 7.5% sodium bicarbonate, penicillin (100 U/mL), streptomycin (100 μg/mL), 200 mM glutamine and 100 μM nonessential amino acids at 30 °C. Human adenocarcinoma (LoVo) cells (obtained from ATCC No. CCL-229) were cultured in Ham’s F-12 medium (Gibco) supplemented with 20% FBS, penicillin (100 U/mL), and streptomycin (100 μg/mL). All cells except for C6/36 were maintained at 37 °C and 5% CO_2_.

DENV2 strain 16681 was kindly provided by Dr. Claire Huang (Center for Disease Control and Prevention, USA). It was propagated in C6/36 cells as described before and called stdDENV^13^. prMDENV particles were produced in LoVo cells, as described previously^13^. Viral infectivity was determined by plaque assay on BHK-21 clone 15 cells (plaque forming units, PFU)^13^. The absolute number of virus particles in solution was determined by quantitative PCR (qPCR), which detects the number of genome-containing particles (GCPs)^17^. Virus infection was performed on the basis of multiplicity of genome-containing particles per cell (MOG).

### Antibodies

Human anti-E (mAb #753 C6) and anti-prM (mAb #3–147) were a generous gift from prof. G. Screaton (Imperial College, London, UK)^18^,^24^. Purified anti-Mouse CD16/CD32 (Mouse BD Fc Block) was obtained from BD Biosciences. Convalescent human dengue immune serum (28 days following DENV2 infection) was generously provided by Dr. G. Comach (Biomed-UC, Lardidev, Maracay, Venezuela) and Dr. T. Kochel (U.S. Naval Medical Research Center Detachment, Lima, Peru). Mouse IgG2A non-DENV Control (anti-KLH) was acquired from R&D Systems. Fab fragments were made from the monoclonal murine anti-E antibody 4G2 (Millipore) by use of the Pierce™ Fab Micro Preparation Kit.

### Drugs and reagents

Endocytotic inhibitors such as chlorpromazine, wortmannin, nystatin, dynasore, AS-604850, cytochalasin B, latrunculin and EIPA were purchased from Sigma Aldrich. Pitstop2 and iminodyn-22 was purchased from Abcam. Ammonium chloride (NH_4_Cl) was obtained from Merck. A furin-specific inhibitor, decanoyl-L-arginyl-L-valyl-L-lysyl-L-arginyl-chloromethylketone (decRRVKR-CMK), was obtained from Calbiochem. The fluorescently labeled endocytic cargos Cholera toxin B-FITC (CtxB-FITC), Dextran-FITC, Dextran-TxRd, and LysotrackerGreen were purchased from Life Technologies.

### Plasmids

The GFP-tagged dominant-negative Eps15 mutant E95/295 and its empty vector D3Δ2 were kindly provided by Dr. A. Benmerah and Dr. A. Dautry-Varsat (Institute Pasteur, Paris, France). The eYFP-Actin construct (human β-actin in a pCDNA3.1 vector) was obtained from Dr. Ben Giepmans (UMCG, Groningen, The Netherlands). The Rab5-S34N-GFP dominant negative mutant (DNM) and Rab5-wt-GFP plasmids were generously provided by Dr. P. van der Sluijs (University Medical Center, Utrecht, The Netherlands). The Rab7-T22N-GFP DNM and Rab7-wt-GFP plasmids were obtained from Gary R. Whittaker (Cornell University College of Veterinary Medicine, NY, USA).

### ADE assay

For virus-antibody complex formation, virus particles (MOG 1000 of std as well as prMDENV) were incubated for 30 min at 37 °C in presence of serial 10-fold dilutions of antibodies in cell culture medium containing 2% FBS. The virus-antibody complexes were then added to 2 × 10^5^ P388D1 cells/well, and incubated at 37 °C with 5% CO_2_. At 43 hours post-infection (hpi), the supernatant was collected and virus particle production was measured with a plaque assay. As controls, an FcR blocker (anti-mouse CD16/CD32) and dynasore were used. The cells were pretreated with the FcR blocker (1 μg per 1 × 10^6^ cells) for 10 min at 4 °C before infection, whereas dynasore (150 μM) was added to the cells 1.5 h before infection.

### DiD-labeling of DENV

The lipophilic fluorescent probe 1,1′-dioctadecyl-3,3,3′,3′-tetramethylindodicarbocyanine, 4-chlorobenzenesulfonate salt (DiD) (Molecular Probes) was used to label the viral membrane of DENV virus particles, as previously described^30^. When incorporated in the viral membrane at a relatively high surface density, the emitted fluorescence level is largely quenched, but single DiD-labeled virus particles can still be clearly detected. Membrane fusion is detected as an increase in fluorescence intensity due to dilution of the probe into the target membrane.

### Microscopic fusion assay

Fusion assays were carried out for DENV in the absence and presence of antibodies. In these assays, the antibody concentration (resp. 400 ng/mL for mAb #753 C6 and 1 ng/mL for mAb # 3–147) that showed maximal enhancement in the infectivity assays was used. Virus antibody opsonization was done as described for the ADE assay. DENV and mAb-DENV complexes were added to P388D1 cells and incubated for 30 min at 37 °C with 5% CO_2_. Then, the cells were washed three times with serum-free, phenol red-free MEM and subjected to microscopic analysis in a Leica Biosystems 6000B instrument. In case of drug inhibitor studies, the cells were prior treated with the drug of interest for the indicated times: Pitstop2 for 10 min; Iminodyn-22 and AS-604850 for 30 min; NH_4_Cl and EIPA for 1 h; chlorpromazine, nystatin, dynasore, cytochalasin B, lantruculin and wortmannin for 1.5 h at 37 °C. An end concentration of 50 mM NH_4_Cl, 25 μM Pitstop2, 15 μM chlorpromazine, 50 μM nystatin, 150 μM dynasore, 200 μM Iminodyn-22, 15 μM cytochalasin B, 1 μM latrunculin, 2 μM wortmannin, 30 μM AS-604850 or 25 μM EIPA was used. All drug dilutions were prepared in serum-free phenol red-free MEM (Gibco) containing 1% glucose and used at non-toxic concentrations. Infection was done in the presence of the compound. Differential interference contrast (DIC) settings were used to randomly select fields. A total of 15 randomly selected fields were taken using both DIC and DiD channels. For the excitation of DiD a 635-nm helium–neon laser was used. The acquired images were processed and analyzed with ImageJ using an in-house macro. The extent of membrane fusion was quantified by measuring the total fluorescent signal per field of view with the “Particle analyzer” plugin of ImageJ. The percentage of fusion inhibition was calculated with respect to the non-treated positive control.

### Single-particle tracking of DiD-DENV

The tracking experiments were performed as previously described^30^. Briefly, P388D1 cells were seeded in 8-wells Lab-Tek II Chambered Coverglass two days before infection. Prior to the experiment, cells were washed three times with phenol red-free MEM and warm phenol red-free MEM containing 1% glucose was added. DiD-labeled DENV (opsonized or non-opsonized std- and prMDENV) was added to P388D1 cells at 37 °C and kept at the same temperature throughout the tracking experiment. DiD-labeled DENV was detected with a 635-nm helium-neon laser. Image series of the fluorescent emission were recorded with a charge-coupled-device camera at 1 frame per s using an epi-fluorescence set-up. Before and after fluorescence imaging, the localization of the nucleus and plasma membrane of the cell was determined by use of DIC optics. The acquired images were processed and analyzed with ImageJ (NIH) and Imaris x64 7.6.1 (Bitplane Scientific Software). Only those virus particles in which the initial fluorescence intensity was below 40 a.u. and that showed more than a two-fold increase in fluorescence intensity upon membrane fusion were used for single-particle tracking analysis. Trajectories were generated by pairing peaks in each frame to previously established trajectories according to proximity and similarity in intensity.

### Electroporation of P388D1 cells

A suspension containing 3×l0^6^ cells (in antibiotic-free DMEM containing 10% FBS) and 10 μg of DNA plasmid were placed in a 0.4 cm electroporation cuvette (Bio-Rad). The electroporation was performed at room temperature, 950 μF and 250 V. Afterwards, the cuvette was kept at room temperature for 4 min. The cells were then resuspended and seeded in an 8-wells Lab-Tek using antibiotic-free 10% FBS DMEM. The media was refreshed one day after electroporation. The cells were used for imaging at two and three days post-electroporation.

### Live-cell imaging in eYFP-Actin expressing cells

P388D1 cells were transfected with the eYFP-Actin construct (10 μg of DNA per 3 × 10^6^ cells) by electroporation and seeded in an 8-wells Lab-Tek II Chambered Coverglass. At 2 days post-electroporation, the cells were washed with serum-free phenol red-free MEM and fresh serum-free phenol red-free 1% glucose MEM was added to the well. Then, DiD-labeled virus (stdDENV or prMDENV) with and without prior antibody opsonization was added *in situ* in the presence or absence of the indicated treatments. The virus used for live-cell imaging was UV-inactivated for 1 h. UV-treatment did not interfere with membrane fusion activity as assessed by the microscopic membrane fusion assay. The eYFP-Actin expressing living P388D1 cells were then imaged with a Solamere Spinning Disk Confocal Live Cell Imaging system (Solamere Technology Group). This system is based on a confocal Leica DM IRE2 Inverted microscope (Leica Microsystems) equipped with a temperature and CO_2_ controlled box (Live Imaging Services). YFP and DiD fluorescence were recorded with a Stanford Photonics XR/Mega-10 ICCD camera (Stanford Photonics) using argon (488 nm) and krypton (647 nm) lasers as light sources, respectively. Time-lapse acquisition was done at 1 image every 4.5 seconds with a 63× objective. Only one focal plane was imaged as analysis in Z-axis drastically reduced the imaging speed. The acquired images were processed and analyzed with Imaris ×64 7.6.1 and ImageJ.

### Fluid phase uptake

P388D1 cells were seeded in 8-wells Lab-Tek II Chambered Coverglass two days before infection. Cells were washed once with phenol red-free MEM and warm phenol red-free MEM containing 1% glucose and either PMA (60 ng/ml; Sigma), stdDENV, Ab-stdDENV or mAb # 753 C6 was added. Cells were incubated for 15 min at 37 °C and subsequently Dextran-TxRd (final concentration: 0.4 mg/ml) was added. Uptake of Dextran was allowed for 30 min at 37 °C, after which cells were washed 3× with warm phenol red-free MEM and fixated with 4% PFA. Differential interference contrast (DIC) settings were used to randomly select fields. A total of 15 randomly selected fields were taken using both DIC and TexasRed channels. The percentage of fluid phase uptake was calculated with respect to the non-treated positive control.

### Dominant-negative mutant assays

DiD-labeled DENV was added *in situ* to P388D1 cells electroporated beforehand with the Eps15-GFP (DNM E95/295 and its empty vector D3Δ2) Rab5-GFP (DNM S34N and WT) and Rab7-GFP (T22N DNM and WT) plasmids. A fusion assay was performed as described in previous sections. To assess the effect of the DNM on membrane fusion of the virus, the extent of membrane fusion was quantified in 85–100 cells positive for the expression of the plasmid (GFP+) as described in previous sections. The percentage of inhibition of fusion of the DNM was calculated with respect to the WT plasmid control.

### Fluorescently labeled cargo controls for biochemical inhibitors and DNM

To confirm the inhibitory activity of the chemical inhibitors, different fluorescently labeled cargo controls were used. The activity of nystatin was confirmed by incubation of cells with CtxB-FITC for 30 min at 37 °C. Cells treated with dynasore, cytochalasin B, latrunculin, wortmannin, AS-604850 and EIPA were incubated with either Dextran-FITC or Dextran-TxRd for 30 min at 37 °C to assess the inhibitory effect. Transferrin-AF633 (15 min at 37 °C) was used as a cargo control for chlorpromazine, pitstop2 and Iminodyn-22. To define the effect of ammonium chloride on the pH of acidic intracellular vesicles, P388D1 cells were stained with LysotrackerGreen for 15 min at 37 °C, then washed twice with serum-free phenol red-free MEM. Visualization was done by use of an epi-fluorescence Leica Biosystems 6000B microscope.

The activity of the Rab5 and Rab7 DNM plasmids was confirmed with the use of Dextran-TxRd. P388D1 cells were electroporated with the Rab5 or Rab7 WT and DNM plasmids. Two days post-electroporation, the cells were treated with Dextran-TxRd (0.03 mg/mL) for 30 min at 37 °C, washed and then imaged to assess Dextran uptake or co-localization. Rab5-electroporated cells were visualized with an epi-fluorescence Leica Biosystems 6000B microscope. To assess the effect of the Rab5-DNM on uptake of Dextran, 50 cells positive for the expression of the plasmid (GFP+) were selected and scored as positive or negative for uptake. The percentage of inhibition of uptake of the DNM was calculated with respect to the WT plasmid control. On the other hand, since expression of the DNM form of Rab7 blocks the exit of cargo molecules from early to late compartments^64^, the activity of the Rab7-DNM was defined by quantifying co-localization of Dextran molecules and Rab7-GFP+ compartments in both Rab7 DMN and wt expressing cells. To this end, the cells were fixed with PFA 4% and imaged with a Leica SP8 Confocal microscope. Fifty Dextran molecules were assessed per condition.

## Additional Information

**How to cite this article**: Ayala-Nunez, N. V. *et al*. How antibodies alter the cell entry pathway of dengue virus particles in macrophages. *Sci. Rep.* **6**, 28768; doi: 10.1038/srep28768 (2016).

## Supplementary Material

Supplementary Information

Supplementary Movie S1

Supplementary Movie S2

Supplementary Movie S3

Supplementary Movie S4

Supplementary Movie S5

Supplementary Movie S6

Supplementary Movie S7

Supplementary Movie S8
