# Supplementary material for: How antibodies alter the cell entry pathway of dengue virus particles in macrophages
Source: Sci Rep. 2016 Jul 7;6:28768. doi: 10.1038/srep28768 (PMC4935958; doi:10.1038/srep28768)
Supplement: Supplementary Information [file srep28768-s1.pdf]

How antibodies alter the cell entry pathway of dengue virus particles in macrophages

Nilda V. Ayala-Nunez[1], Tabitha E. Hoornweg[1], Denise P.I. van de Pol[1], Klaas A. Sjollema[2], Jacky Flipse, Hilde van der Schaar[3], Jolanda M. Smit[1*]

[1] Dept. of Medical Microbiology, University of Groningen, University Medical Center Groningen, Groningen, The Netherlands

[2] Dept. of Cell Biology, University of Groningen, University Medical Center Groningen, Groningen, The Netherlands

[3] Dept. of Infectious Diseases & Immunology, Virology Division, Faculty of Veterinary Medicine, Utrecht University, Utrecht, The Netherlands

*Contact Information:

jolanda.smit@umcg.nl

Tel: +31 50 363 2738

Supplementary information

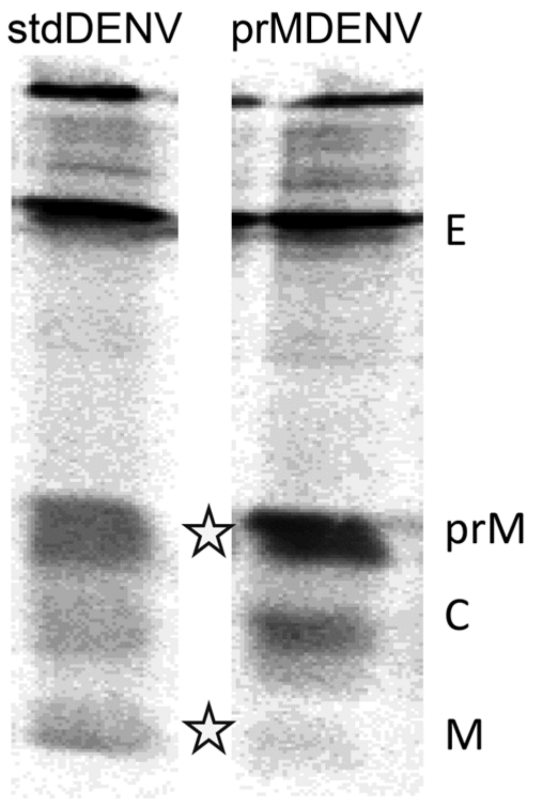

Figure S1. Representative image of purified [35S]methionine-labeled standard and immature DENV particles. 35S-labeled DENV was prepared as described before [13]. Protein composition was analyzed by non-reducing SDS-PAGE gel electrophoresis. Quantification of the protein bands was done by phosphorimaging analysis using ImageQuant TL software. The percentage of prM and M in virions was determined by relating the intensity of the prM and M bands to that of E, on the basis of the relative number of methionine residues in each protein and with the assumption of uniform labeling. LoVo-derived DENV has on average 94% +/- 9% in the viral membrane[13].

PFUs/ml	GCPs/ml	Ratio
6,00E+02	2,91E+10	4,84E+07
1,00E+02	1,81E+10	1,81E+08
1,20E+03	3,69E+10	3,08E+07
3,00E+02	3,04E+10	1,01E+08
1,20E+03	2,75E+10	2,29E+07
1,80E+03	6,16E+10	3,42E+07

Table S1. Purified LoVo-derived DENV, characteristics of the individuals preparations. The average ratio of these preparations is 6,98E+07.

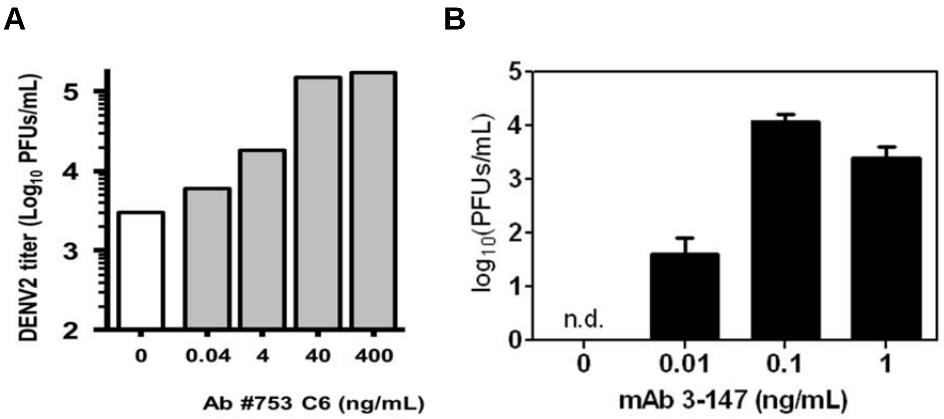

Figure S2. Anti-E and anti-prM antibodies enhance DENV infection via FcγRs. (A) Enhancing profile of anti-E mAb 753 C6 complexed to stdDENV in P388D1 cells. n (B) Enhancing profile of anti-prM mAb 3-147 complexed to prMDENV. Virus particle production was assessed at 43 hours post-infection by plaque assay. Limit of detection: $6*10^1$ PFUs/ml. N.d. denotes not detectable.

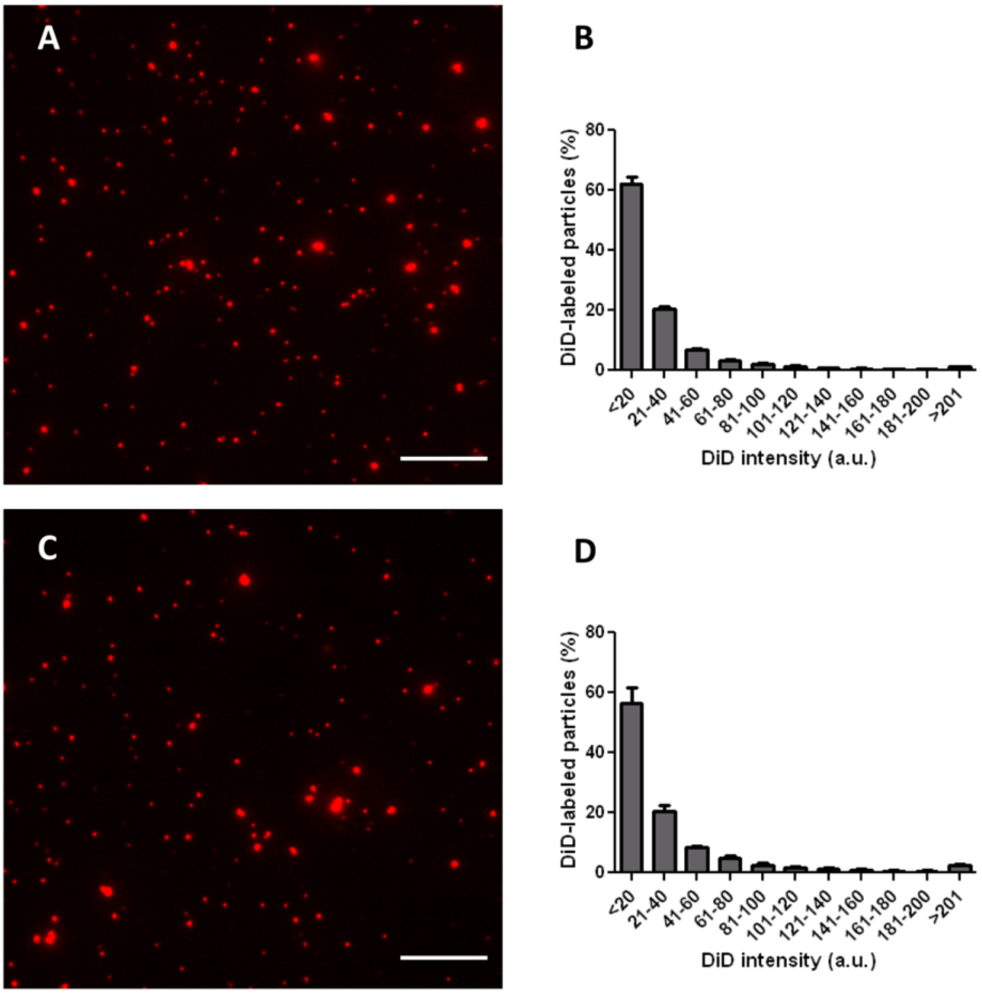

Figure S3. Size distribution of DiD-labeled DENV. stdDENV was labeled with DiD as described in materials and methods. After labeling the virus was either directly visualized by microscopy (A,B; stdDENV) or opsonized with Ab 735 C6 (final concentration of 40 ng/ml) for 30 min at 37°C previous to visualization (C,D; Ab-stdDENV). A & C show two representative images of DiD-labeled stdDENV and Ab-stdDENV, respectively. Scale bar represents 10 μm. B & D Histograms of the DiD-intensity of stdDENV and Ab-stdDENV, respectively. 3 independent experiments were performed per condition, imaging was done with >5000 particles per experiment. Error bars represent SD.

Methods: The intensity of individual and antibody-opsonized DiD-labeled DENV particles was estimated by fluorescence microscopy as described previously (Van der Schaar, 2007). stdDENV was opsonized using mAb #735 C6 (final concentration 40 ng/ml) for 30 min at 37°C. DiD-labeled stdDENV and Ab-stdDENV were visualized by epi-fluorescence microscopy in a Leica Biosystem 6000B instrument using a 635 nm helium-neon laser. Analysis was done using the 'particle analyzer' plugin of ImageJ.

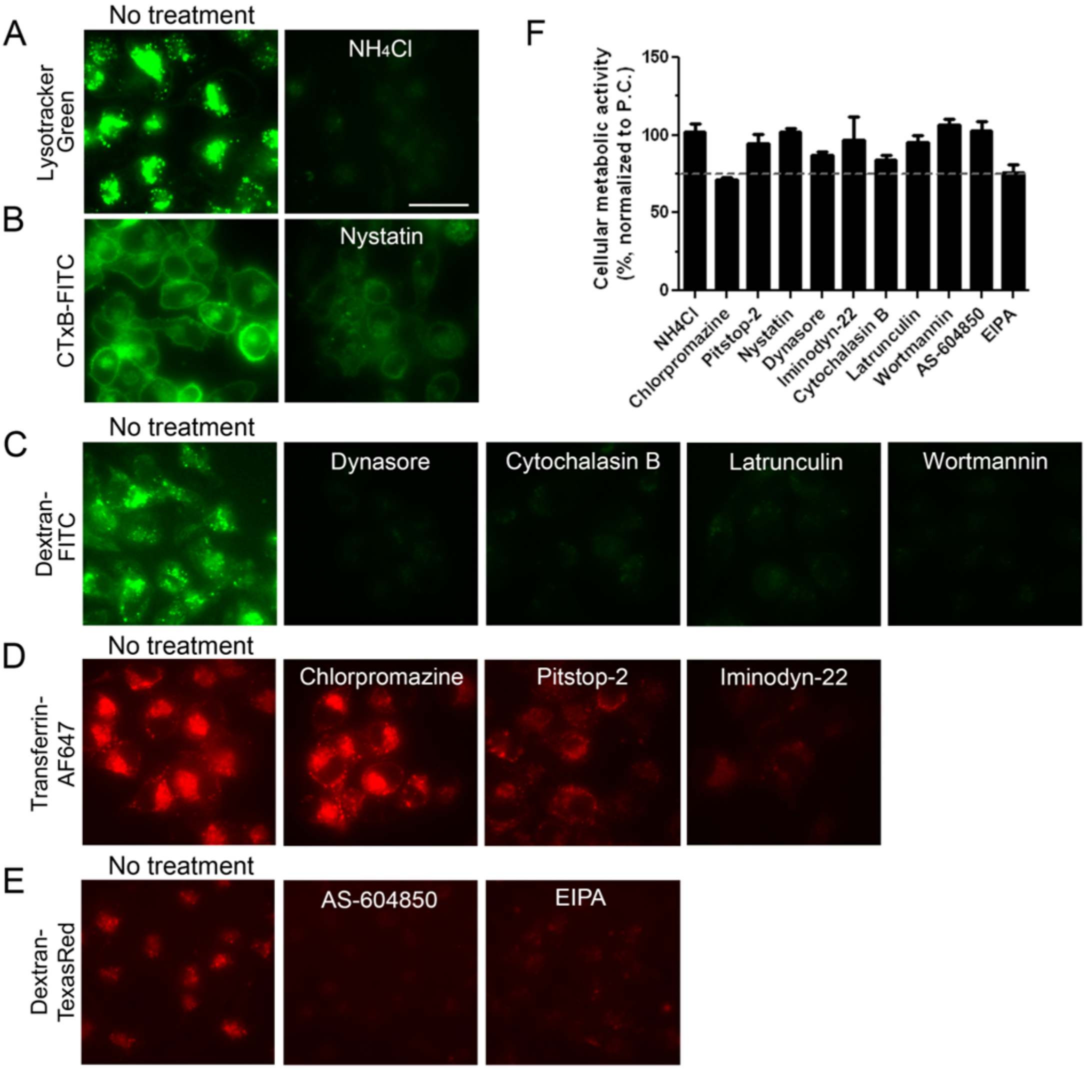

Figure S4. Fluorescently labeled cargo controls and MTT viability assay used to confirm the activity of different biochemical inhibitors. The cargo controls were added to P388D1 cells upon treatment with biochemical inhibitors. (A) Lysotracker Green was used for ammonium chloride (50 mM), (B) CTxB-FITC for nystatin (50 µM). (C & E) Dextran-FITC or Dextran-TxRd for dynasore (150 µM), cytochalasin B (15 µM), latrunculin (1 µM), wortmannin (2 µM), AS-604850 (30 µM) and EIPA (25 µM). (D) Transferrin-AF633 for chlorpromazine (15 µM), Pitstop2 (25 µM) and Iminodyn-22 (200 µM). Scale bar: 25 µm. (F) Standard MTT assay to test cell viability upon treatment with biochemical inhibitors. Using an MTT assay the cellular metabolic activity is tested, which is used as an indicator for cell viability. The grey dashed line indicates 75% cell metabolic activity.

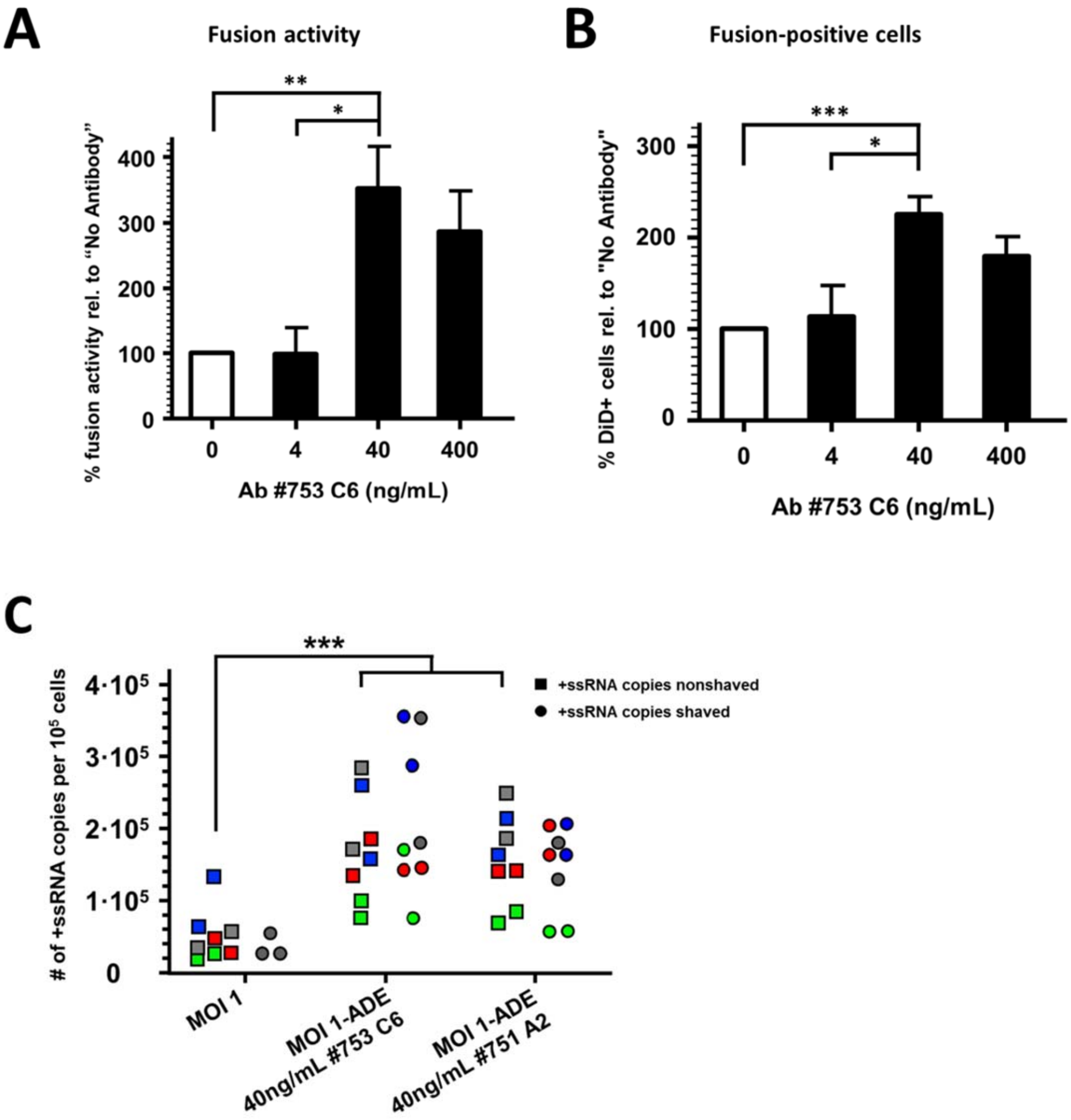

Figure S5. Antibodies promote DENV2 cell entry into P338D1 cells. (A – B) The extent of membrane fusion of DENV2 within P338D1 cells was determined 30 min post-infection at MOI 1. (A) Total fusion activity (B) Fraction of fusion-positive cells (B). All values were normalized to infection in absence of antibodies. (C) DENV2 binding and uptake in P388D1 cells was determined at 1hpi by qRT-PCR using template-specific primers in combination with RNAse A treatment. Extracellular virus was removed by shaving the cells with a high-salt-high-pH buffer for 2 min. Ab #751 A2 is a DENV E antibody that interacts with the

fusion loop in DII [24]. Squares show the total number of virus particles that had bound or entered cells. Circles depict entered viral genomes. The colors indicate replicate samples of independent experiments. Statistical analysis was done by 2-tailed t-test; * ($P \leq 0.05$), ** ($P \leq 0.01$), *** ($P \leq 0.0005$)

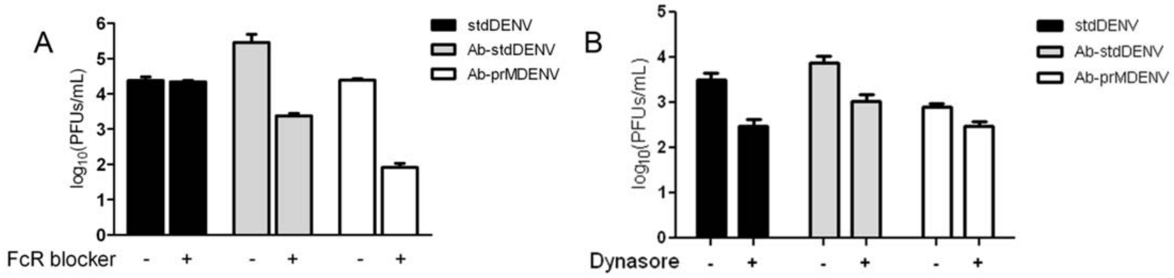

Figure S6. DENV particle production in cells treated with FcR blocker or Dynasore. (A) PFU production of stdDENV, Ab+stdDENV and Ab+prMDENV in presence or absence of FcR Blocker. (B) PFU production of stdDENV, Ab+stdDENV and Ab+prMDENV in presence or absence of Dynasore. Bars represent the average of three independent experiments ± SEM. Limit of detection: $6*10^1$ PFUs/ml.

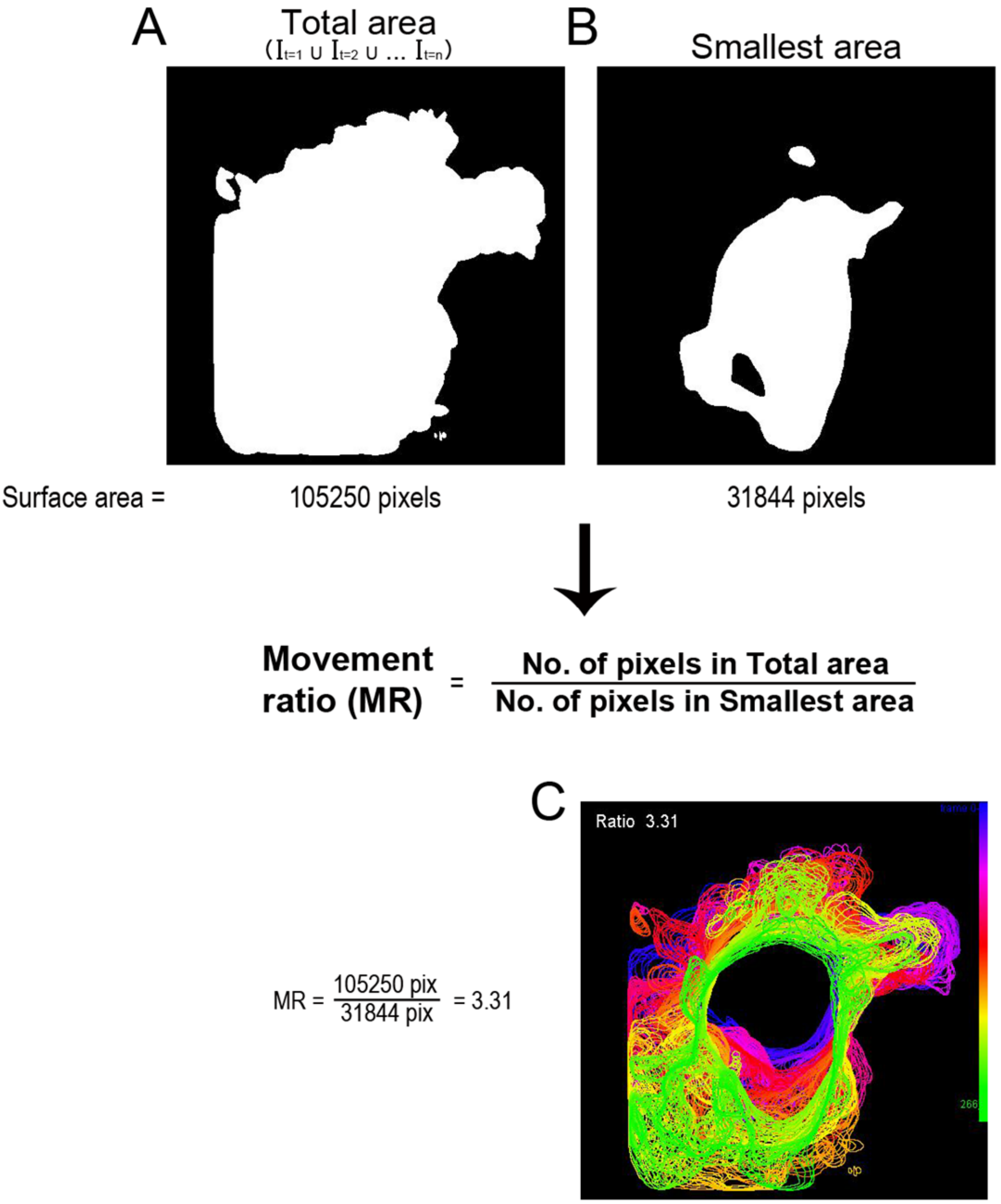

Figure S7. How to calculate the "Movement ratio" (MR). The Movement ratio quantifies the extent of movement of one cell over a frame sequence. To calculate it, images from the sequence are thresholded (binarized) to identify the pixels occupied by the cell at each frame. (A) The "total area" covered by the moving cell for the whole sequence is obtained by counting all pixels in the union of these binarized frames. (B) The area occupied by the cell

before movement starts (smallest area) is obtained from the binarized frame with the smallest number of pixels. Finally, the MR in the image sequence is quantified by the ratio of the pixels obtained in (A) and (B), where 1 corresponds to immobility and higher numbers correlate with higher cell mobility. In this example, we used a sequence of 266 frames. (C) Overlay of the cell body outlines of the same time-lapse. The colors indicate time direction. The whole procedure was done with ImageJ.

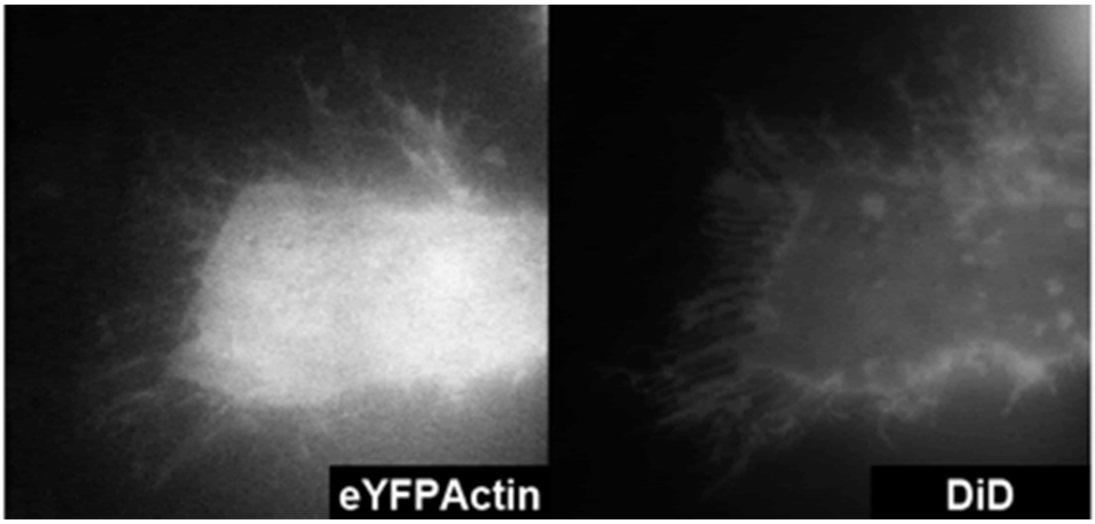

Figure S8. Overlap of eYFP-Actin expression and DiD-labeling of cytoplasmic membrane. P388D1 cells electroporated with eYFP-Actin were stained with the lipophilic probe DiD for 1 h at 37ºC. DiD was incorporated on the cell membrane. The cells were washed and imaged with an epi-fluorescence Leica Biosystems 6000B microscope. As shown in the figure, the DiD signal overlaps with eYFP-Actin.

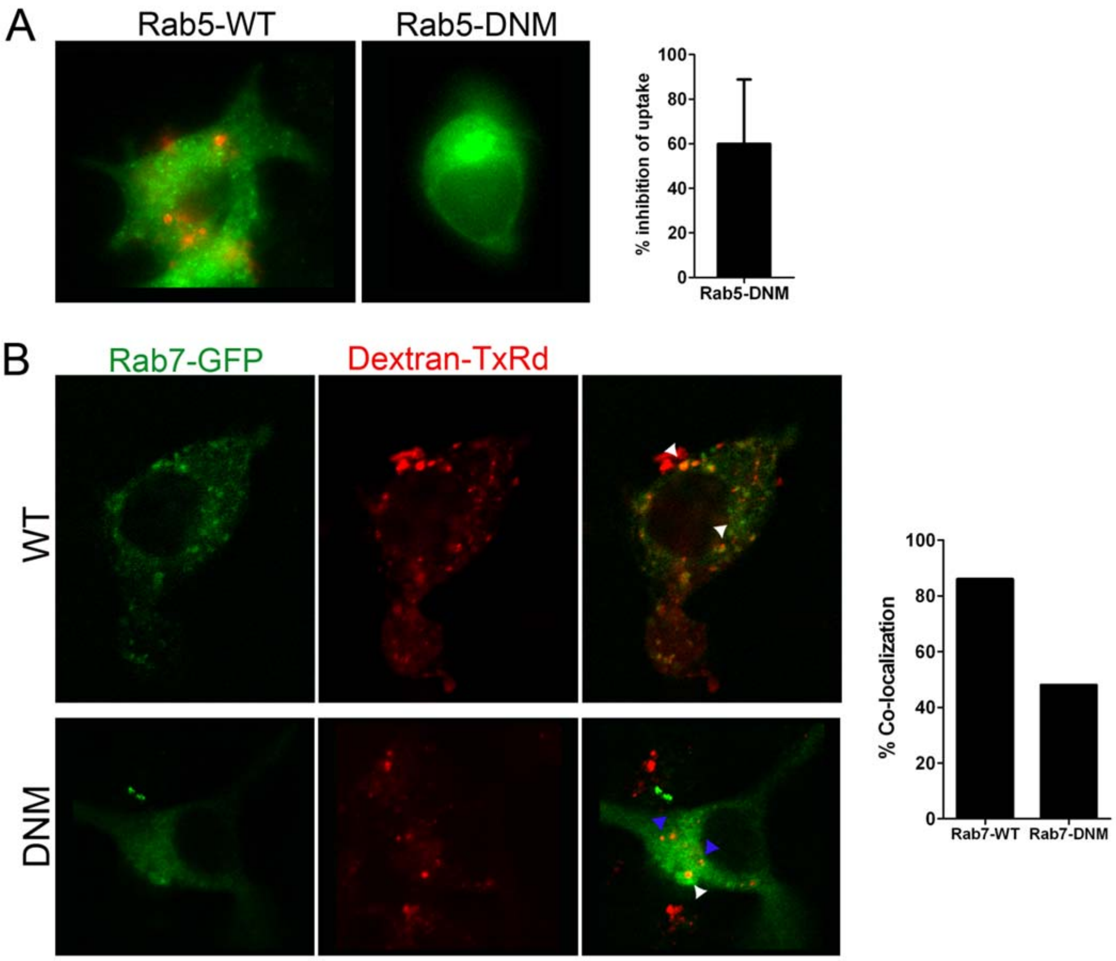

Figure S9. Fluorescently labeled cargo controls to confirm the activity of Rab5-DNM and Rab7-DNM

(A) Dextran-TxRd was added to P388D1 cells after electroporation with Rab5 or Rab7 plasmids (GFP-tagged WT and DNM). The activity of the Rab5-DNM was assessed by quantifying Dextran-TxRd uptake in the DNM and WT cells. To this end, 50 cells were used of each condition. The percentage of inhibition of uptake of the DNM was calculated with respect to the WT plasmid control. (B) The activity of the Rab7-DNM was defined by quantifying co-localization of Dextran-TxRd molecules and Rab7-GFP+ compartments. The white arrowheads indicate examples of co-localization. The blue arrowheads show examples of what was scored as no co-localization. The graphs shows co-localization results of 50 Dextran molecules per condition.

Movie S1. Single-particle tracking of Ab-stdDENV particle in P388D1 cells. DiD-labeled stdDENV particles were opsonized with mAb5 and added *in situ* to the cells. The movie was recorded at a time-lapse of 1 frame per second using an epi-fluorescence Leica Biosystems 6000B microscope. A sequence of 70 frames is shown. Membrane fusion is evident as a sudden increase in DiD fluorescence intensity. The images were artificially modified to show the differences in fluorescence intensity, from low intensity (purple) to high intensity (yellow).

Movie S2. Extensive ruffling induced by Ab-stdDENV. Actin-eYFP expressing P388D1 cells were infected with DiD-labeled Ab-stdDENV. The cells were then imaged with a Solamere Spinning Disk Confocal Live Cell Imaging system at 1 frame per 4.5 seconds with a 63x objective. Shown is a sequence of 300 frames. For clarity, the DiD channel is not shown.

Movie S3. Extensive ruffling induced by Ab-prMDENV. Actin-eYFP expressing P388D1 cells were infected with DiD-labeled Ab-prMDENV. Imaging was performed as described to the legend of Movie S2. A sequence of 400 frames is shown. The DiD channel is not shown for clarity purposes.

Movie S4. Mock-infected P388D1 cells. Actin-eYFP expressing P388D1 cells were incubated with HNE buffer and imaged as described to the legend of Movie S2. A sequence of 250 frames is shown.

Movie S5. Cells before Ab-DENV induced extensive ruffling. Actin-eYFP expressing cells were infected with DiD-labeled Ab-prMDENV. Imaging was performed as described to the legend of Movie S2. The movie shows the first minutes following infection.

Movie S6. Cells after Ab-DENV induced extensive ruffling. Actin-eYFP expressing cells were infected with DiD-labeled Ab-prMDENV. Imaging was performed as described to the legend of Movie S2. The movie starts at 4.5 mpi. A sequence of 160 frames is shown.

Movie S7. Example of Type 2 uptake. Actin-eYFP expressing P388D1 cells were infected with DiD-labeled Ab-prMDENV. Imaging was performed as described to the legend of Movie S2. A sequence of 16 frames is shown.

Movie S8. Example of a multiple Type 2 uptake attempts to capture the virion. Actin-eYFP expressing P388D1 cells were infected with DiD-labeled Ab-stdDENV. Imaging was performed as described to the legend of Movie S2. A sequence of 300 frames is shown.
